# Antimalarial efficacy of aqueous crude rootbark extracts of *Zanthoxylum Chalybeum: An in vivo* experimental study in Wistar Albino Mice infected with *Plasmodium berghei*

**DOI:** 10.21203/rs.3.rs-8627334/v1

**Published:** 2026-01-20

**Authors:** Okot Michael, Catherine Nassozi Lwanira, Moses Ocan, Paul E. Alele

**Affiliations:** Department of Toxicology, School of Pharmacy, University of Eastern Finland. P.O Box 1627, FI-70211, Kuopio, Finland; Department of Biochemistry and Systems Biology, College of Natural Sciences, Makerere University, P.O Box 7062, Kampala, Uganda; Department of Pharmacology and Therapeutics, College of Health Sciences, Makerere University, P.O Box 7072, Kampala, Uganda; Department of Pharmacology and Therapeutics, Mbarara University of Science and Technology, P.O Box 1410, Mbarara, Uganda

**Keywords:** Malaria, Zanthoxylum chalybeum, Plasmodium berghei, Parasite clearance

## Abstract

**Background:**

Malaria remains a significant cause of morbidity and mortality globally. Previous *in vitro* studies have demonstrated potential anti-plasmodial activity of *Zanthoxylum chalybeum*. We thus carried out an *in vivo* study to determine the efficacy of *Z.chalybeum* on parasite clearance, haemoglobin preservation, and fever reduction in Wistar albino mice infected with *Plasmodium berghei NK65.*

**Methods:**

This was an *in vivo* experimental study to determine the curative plasmodial clearance of *Z.chalybeum* in Wistar albino mice infected with *Plasmodium berghei NK65.* The mice were cage-housed in three groups; the experimental group A consisting of subgroups A1, A2 and A3 was administered with *Z.chalybeum* aqueous rootbark extracts at concentrations of 400mg/kg, 200mg/kg, and 100mg/kg respectively. The positive control (B) received Artemether-Lumefantrine at 4mg/kg body weight, and group C (the negative control) was given distilled water used to dilute the rootbark extracts at a dose of 10mL/kg. The treatment period was five days beginning from day three post-infection of the experimental mice. Differences in parasite clearance, hemoglobin concentration and fever reduction among the experimental groups were analyzed using one way analysis of variance (ANOVA). P-values less than 0.05 were considered to be statistically significant.

**Results:**

*Z. chalybeum* aqueous rootbark extracts showed parasite clearance up to 76% in Wistar albino mice infected with *P. berghei.* Compared to the negative control group, the root bark extracts exhibited significantly higher parasite clearance at 100 mg/kg dose (mean difference (MD) = 15.6, 95%CI = 14.95–16.25; p < 0.001), 200 mg/kg (MD = 16.42, 95%CI = 15.77–17.07; p < 0.001) and 400 mg/kg (MD = 17.24, 95%CI = 16.59–17.89, p < 0.001). Further, the mean temperature difference was found to be significantly lower among the experimental group as compared to the negative control group which was, MD=−1.50, 95%CI = 2.59–0.67; p = 0.009 at 100mg/kg dose; MD=−1.63, 95%CI = 2.74–0.81; p = 0.004 at 200mg/kg and MD=−1.78, 95%CI = 2.46–0.54; p = 0.002 at 400mg/kg of administered dose. There were no remarkable differences between the haemoglobin and haematocrit concentrations of the experimental and negative control groups.

**Conclusion:**

*Z. chalybeum* showed efficacy in parasite clearance and maintaining the body temperature of Wistar albino mice infected with *P. berghei*. Additional studies should be done to determine the anti-plasmodial and anti-pyretic mechanisms of *Z. chalybeum* root bark extracts.

## Background

Malaria remains a significant cause of morbidity and mortality especially among children under five years and pregnant mothers [1]. Globally, malaria accounted for an estimated 249 million cases and 608, 000 deaths in 2022. The majority of these cases (94%) were found in the African region, where almost half of all cases were contributed by four countries – Nigeria (27%), the Democratic Republic of the Congo (12%), Uganda (5%) and Mozambique (4%) [1]. Whereas available preventive and curative strategies have been remarkable in reducing malaria-associated mortality, they continue to be under constant threat by parasites and vectors that develop resistance to anti-malarial drugs [2–4] and insecticides [5–8]. Thus, continuous evaluation of potential anti-malarial drugs is warranted among other promising strategies for protection of individuals against malaria.

Over the years, anti-malarial treatment strategies have been largely based on conventional therapy. However, these remain challenged due to the increasing trends in resistance to current conventional antimalarials by the *Plasmodium;* notably, Chloroquine [9], Mefloquine [10], Quinine [11], Sulfadoxine/Pyrimethamine [12] and Atovaquone [13]. Additionally, conventional antimalarial drugs come along with some adverse effects and high costs of treatment amounting to an average of USD 5 for a single dose of artemisinin combination therapy (ACT) used as first line treatment in Uganda [14]. This makes the local community unable to afford and or even tolerate the side effects of commonly used conventional drug regimens.

The Global Technical Strategy for malaria prevention aimed at reducing incidence and mortality associated with malaria infection by 40% before 2020 [15]. However, this has not been achieved due to the lack of comprehensive approaches of management of malaria including inappropriate use of malarial herbal medicine without evidence to prove their effectiveness in the management of malaria [15]. This necessitates research on malarial medicinal plants to build strong evidence on their use as a remedy for malaria. *Zanthoxylum. chalybeum* (Knob tree), identified in other local languages as “Eusuk” (in Ateso), “Ekibirizi” (in Rutooro), “Ntaleye ddungu” (in Luganda) “Kichuk roki” (in Luo) and “Pupwechulu pupwe (in Bemba) is a shrub that has been used for decades as a remedy for malaria among indigenous communities of Uganda. Previous studies have demonstrated *in vitro* assessment anti-plasmodial activity of *Z. chalybeum* stem bark against chloroquine-sensitive (NF54) and chloroquine-resistant (FCR3) *P. falciparum* strains [16], while also its cytotoxicity and acute toxicity was shown [17]. In another study, moderate *in vitro* anti-plasmodial activity (IC_50_ values of 11–50 *μ*g/ml) was observed for all the extracts of *Z. chalybeum* leaves used to treat malaria in Embu County, Kenya [18]. Results from a systematic review of studies in across Africa have consistently confirmed good anti-plasmodial activity of *Z. chalybeum* [19]. *Z. chalybeum* contains bioactive compounds including fagaramide, tembetarine, and other alkaloids such as nitidine, chelerythrine and skimmianine that are thought to contribute towards its antimalarial activity [20].

*Z. chalybeum* rootbark extracts are widely used for malaria treatment among indigenous communities of Northeastern Uganda [21]. Despite the promising *in vitro* activity of *Z. chalybeum*, its replicability to *in vivo* studies may not easily be translated. Therefore, we conducted an *in vivo* experimental study to examine the efficacy of *Z. chalybeum* (“Eusuk”) rootbark extracts on parasite levels, fever reduction, and haemoglobin preservation (reduction of hemolysis) in Wistar albino mice infected with *P. berghei.*

## Materials and Methods

### Study design and setting

We evaluated the efficacy of *Z. chalybeum* (“Eusuk”) rootbark extracts on parasite levels, fever reduction, and haemoglobin preservation (reduction of haemolysis) in Wistar albino mice infected with *P. berghei.* The study was conducted at Mbarara University of Science and Technology (MUST) Animal Research and Pharmacology/Pharmacy laboratories. At the animal research laboratory, mice were obtained and preserved under standard conditions as per guidelines provided by the Uganda National Council of Science and Technology (UNCST) [22]. Other laboratory investigations carried out in this laboratory were drug administration and sample measurements. Drying, extraction, storage of the plant material and microscopy for the quantification of percentage parasitaemia were done at the Pharmacology/Pharmacy laboratory.

### Plant material

Fresh rootbark parts of *Z. chalybeum* were harvested from Olilim village, Olilim Parish, Nyero subcounty, Kumi district in December 2022. The plant materials were harvested during rainy season with the help of a traditional healer. Thereafter, the materials were transported covered in aluminum paper to prevent exposure to sunlight to MUST Pharmacology Laboratory. The sample was identified by a botanist and the specimens deposited at the Herbarium of Makerere University College of Health Sciences.

#### Preparation of the test extract of Z. chalybeum

Fresh rootbarks of *Z. chalybeum* were debarked and air-dried at the MUST Pharmacology Laboratory for two weeks. The root barks were mechanically pulverized using RRH-1000 High-speed multifunction comminutor electric mortar at voltage of 220 v-240 v 50/60 hz, power of 2800W at the speed of 25000 r/min and degree of grinding of 50–300 mesh to form yellowish fine powder. A portion of the powder (100 g) was dissolved in distilled water at a ratio of 1:10 and the mixture was boiled for 30 minutes to enhance the dissolution of the active compounds from the lignified rootbarks. This method was chosen to resemble the method used by the local community, where the dried powder of *Z. chalybeum* rootbarks is mixed with hot water to form a decoction. After boiling, the decoction was filtered using filter paper Whatman cat No. 1440150 to form a clear yellowish decoction measuring 700 ml. The decoction was then frozen using deep freezer model 2180028 LLEXICON, UUS-552–1 at −86°C for 72hours. The deeply frozen extract was transferred to the vacuum bench top model FDI-KL for dehydrating. The viscous solid formed was air-dried for 72 hours to obtain a dry extract that weighed 23g. The percentage extraction (23%) was determined by dividing 23g/100g multiplied by 100%. The powdered extract was transferred into a tight seal opaque container, labeled and stored at + 2°C in a refrigerator model MPR-514PE ready for use. The remaining root bark powder was transferred in a separate tight seal container and stored at room temperature in the Pharmacology Laboratory for future use.

### Animal handling and sample size estimation

Wistar albino mice were obtained from the Animal Research Laboratory, Department of Pharmacology at MUST. The mice were grouped and kept in the animal laboratory at 25–27°C with 12 hours of natural light. The animals were allowed to have free access to food, feeding on commercial pellets and water for a period of seven days in order for them to acclimatize [23]. All the procedures of handling the mice were carried out according to the UNSCT guidelines on research involving animal handling [24].

The sample size for the mice was determined using the E formula [25]. A total of 36 mice were used for the study including one additional *P. berghei* NK65 donor mouse and seven healthy mice for determining lethal dose at 50 (LD50).

### Parasite and infection procedures

After mice had acclimatized, they were inoculated with *P. berghei* N65 and allowed to stay for another 72hours. The infection species of *P. berghei NK65* was obtained from MUST Pharmacology Laboratory. The preparation contained 0.5 millilitres (ml) of *P. berghei NK65*, mode ATCC MRA-269 *P. berghei NK65CS* (−) parasite, Lot No.2082169 infected mouse blood in glycerate 57 solution in a ratio of 1:2. The product vial was packaged aseptically in cryovials and stored at −80°C throughout the study. The study used one mouse as a donor for inoculation which was infected following guidelines as previously described [26]. The frozen cryovial of MRA-268 was thawed in a 35°C water bath for 3 minutes while not allowing the cap line seal of the vial to immerse. After thawing, the surface of the vial was wiped with 70% ethanol before opening to disinfect it. Using a one-ml syringe equipped with 27-gauge ½ inch needle, 0.2ml of mouse infected blood was removed from the vial. The injection site of the mouse was wiped with 70% ethanol and the sample (100 μl) was injected intraperitoneally at the lateral aspect near the hindlimb.

### Monitoring parasitaemia

Parasitaemia was detected by microscopy starting 72 hours after inoculation. As previously described [27], blood smears were prepared in three quarters of the microscopic slide and allowed to air dry for 30 minutes. After, the microscopic smeared slide was then washed with dehydrated menthol (99%) to fix the parasites and thereafter allowed to air dry for 20 minutes. The fixed microscopic slides were then stained with 10% Giemsa stain which was freshly prepared by obtaining 1ml of 3.8g in 500ml stock solution of Giemsa and mixing it with 9mls of distilled water. The stained microscopic slide was allowed to air dry for 30 minutes and later observed under the binocular microscopic in X100 objective lens using immersion oil. The percentage parasitaemia was obtained by counting the number of parasitised red blood cells and the normal red blood cells in 5 five microscopic fields. The percentage parasitaemia that was obtained in the donor mouse was 21.2% after 72 hours of inoculation [26].

### Quality control for Microscopy

Methanol (99%) was used to fixed *P. berghei* in pre-dried slides by washing the slides in methanol for three minutes. A giemsa stock 10% (GURR giemsa staining solution, VWR chemical, PROLABO; Europe C) which was obtained from Pharmacology laboratory was used to prepare a fresh stock solution for staining. Buffered distilled water (PH 7.2) was used as a diluting solution and washing of the controlled slides was done for 20 minutes. The slides were then air dried for twelve hours ready for microscopic observation. The parasitaemia level was quantified by observing the number of parasitized and nonparasitized red blood cells. For consistency of results, five microscopic fields were observed in each microscopic slide examined and for the accuracy in reporting, a certified microscopy performed initial examination of each microscopic slide and the second option was given by the researcher. Before the examination was done, the microscope was calibrated by using *P. falciparum* pre-prepared microscopic slide which was examined by the two certified microscopists.

### Passage of parasites to experimental animals

The donor infected mouse with a known 21.2% parasitaemia level was transferred into an anaesthetic jar treated with 2ml of halothane and allowed to stay in the jar for 5 minutes until immobile. The mouse was then moved onto a dissecting board, where an incision was made through the rib cage to expose the heart. Using a 27-gauge ½ inch needle, 0.25ml of blood was drawn as predetermined from the following formula as previously described [26].

$$\text{Amount}\;\text{of}\;\text{mouse}\;\text{blood}\;\text{to}\;\text{be}\;\text{obtained}=\frac{30\;x0.2\text{ml}}{90x22.21/100}$$

Where, 30 is the number of mice to be inoculated, 0.2ml is amount used for each inoculum. Each mouse received 200μg/ml of parasite, 90 was the dilution factor and 22.21 was the percentage parasitaemia. Accordingly, 0.2ml of blood was taken from the 5 ml mouse normal saline at ratio of 0.3 ml: 4.7 ml and inoculated to each study mouse by intraperitoneal injection at lateral aspect near the hind limb. This position was adopted to avoid injuring the liver or injecting inoculum directly into the liver.

### Treatment of experimental Mice

After 72 hours of inoculation of the experimental mice with *P. berghei NK6*, the animals were randomized into different groups and received treatment as follows: Group A experimental group received different concentrations of *Z. chalybeum* root bark extracts at 100 mg/kg, 200 mg/kg and 400mg/kg in groups labeled as A1, A2 and A3, respectively. The initial dose determination was guided by up and down method with consideration of LD50 which was > 2000mg/kg. The LD50 was divided by five days to obtain the maximum initial dose of 400mg/kg considering not to exceed the LD50 in five-day dosing among the study group that received the highest concentration of the extracts. The dose was then halved to get moderate and lower dose respectively. Group B was the positive experimental group and received artemether lumefantrine (Lonart) at 4 mg/kg body weight. This dose was determined by mixing 140 mg of Lonart with 20 ml of distilled water to form a concentration of 1mg/kg. Group C was a negative experimental group and received distilled water which was used to dilute *Z. chalybeum* rootbark extracts at 10 ml/kg. The dosing was done daily for; 1, 2, 3, 4 and 5 days.

The volume of the products to be administered to experimental mice was determined using the formula described by Girma et al., [28] as;

$$\text{Volume}=\frac{\;\text{Dose}\;\text{in}\;\text{mglkg}\;x\;\text{Weight}\;(\text{kg})}{\text{Concentration}\;\text{in}\;\text{mg}/\text{ml}}$$

#### Acute oral toxicity testing of Z. chalybeum rootbark extracts

An acute oral toxicity test of *Z. chalybeum* rootbark extracts was performed on seven randomly selected non-infected female mice following the Organization for Economic and Development (OECD) guidelines 425. The mice with average weight of 19.5g were fasted overnight and weighed before the test. The recommended dose progression was determined as 5.5 mg/kg, 17.5 mg/kg, 175 mg/kg, 550 mg/kg and 2000 mg/kg using the OECD software. The initial administered dose was selected at 17.5 mg/kg and the extract were given orally for seven days after 24 hours apart of observation. For the accuracy of the observation, it was done using Canon digital camera. The signs of toxicity were noted by either the death of mice in a given dose and was recorded as “X” or if no mice died after 24 hours in a given dose, was recorded as “0”. The maximum dose where no mice died was noted. This dose was repeated for another 2 days and still no mice died. The LD50 was determined using AOT425 software version 1.0.

### Measurements of basic parameters

#### Temperature measurement

The temperature of the mice was measured using a rectal digital thermometer. For consistency of reading, a proven digital thermometer calibrated by the temperature of the controlled non-experimental group was inserted in the anus of the hand-held mouse and the thermometer inserted in the mouse rectum. The temperature of the mouse was automatically read after one minute. This was done for all the experimental mice on day one of initiation of administration of the *Z. chalybeum* rootbark extract until day five of end of administration of the extracts. The results were recorded in a data monitoring tool.

#### Detection of parasitaemia

Parasitaemia was detected by microscopy as previously described by Bailey et al., [27]. The percentage parasitaemia was determined by observing five microscopic fields while counting the number of parasitized red blood cells and the total number of the red blood cells in the field. This was done by dividing the microscopic field into ¼ and using the hand-held counter, parasitized red blood cells and total red blood cells were counted. The percentage parasitaemia was determined as described elsewhere [29] using the formula:

$$\text{Percentage}\;\text{parasitemia}=\frac{\text{Total}\;\;\text{number}\;\text{of}\;\text{parasitied}\;\text{red}\;\text{blood}\;\text{cells}\;\text{in}\;5\;\text{microscopic}\;\text{fields}}{\text{Total}\;\;\text{number}\;\text{of}\;\text{red}\;\text{blood}\;\text{cellso}\;\text{bserved}\;\text{in}\;5\;\text{microscopic}\;\text{fields}}\times 100$$

The percentage clearance of each fraction was compared in respect to the controls and the percentageparasitaemia suppression was calculated using the formula described below [30]:

$$\text{Percentage}\;\text{clearance}=\frac{\text{Parasitemia}\;\text{of}\;\text{the}\;\text{negative}\;\text{control}-\text{parasitemia}\;\text{of}\;\text{the}\;\text{treated}\;\text{group}}{\;\text{mean}\;\text{parasitemia}\;\text{of}\;\text{the}\;\text{negative}\;\text{group}}x100$$

#### Measurement of hemoglobin and haematocrit

The haemoglobin (Hb) and haematocrit (HCT) of the mice was measured using Misson plus Hb haemoglobin testing system Lot HbM2020015, LCA628203 (ACON laboratories, inc. 5850 Oberlin drive 340 San Diego, CA2121, USA). The Hb meter was calibrated by measuring the Hb and HCT of five health non experimental mice which also gave a controlled reference Hb and HCT levels to be extrapolated in the experimental mice. After inserting the strip code on the machine, a minor cut was made on each mouse tail to access the blood from the tail vein. A drop of blood (0.5μl) was put on the strip, inserted on the haemoglobin machine using pipette, and the result was automatically read after 15 seconds and recorded on the data monitoring tool. This was performed daily for the whole treatment period of five days.

#### Determination of mean survival time (MST)

Survival time was recorded to determine the effectiveness of *Z. chalybeum* aqueous rootbark extracts on twenty-eight-day survival of mice infected with *P. berghei* NK65. The mice were observed for 28 days from the time of inoculation, and death that occurred during this period was recorded in experimental groups. The survival rate was expressed as survival function (S) a formula by Kaplan Meier survival estimates [31]:

$$\text{MST}=\frac{\text{Number}\;\text{of}\;\text{individual}\;\text{mice}\;\text{surviving}\;\text{longer}\;\text{for}\;28\;\text{days}}{\text{total}\;\text{number}\;\text{of}\;\text{individual}\;\text{mice}\;\text{studied}}$$

#### Statistical analysis

Data were analyzed using GraphPad prism version 6.0. The results were presented in tables as means and standard error of means. Inferential statistics that included weight, temperature, percentage parasitemia, hemoglobin and hematocrit were performed using one-way analysis of variance (ANOVA) and mean differences of measured parameters were compared using Tukey’s multiple comparison test. For all statistical tests, a P value less than 0.05 was taken as statistically significant.

## Results

A total of thirty-six mice were used for testing the efficacy of *Z. chalybeum* anti-plasmodial clearance. Six mice were used in each test group comprising of the negative control, positive control and the experimental groups which received 100mg/kg, 200mg/kg and 400mg/kg of the aqueous root bark extract of *Z. chalybeum*. Additional six mice were used in an acute toxicity study to determine the LD50 of the extracts. *Z. chalybeum* was found to have LD50 > 2000mg/kg.

### Efficacy of Z. chalybeum aqueous rootbark extracts on parasite clearance in Wistar albino mice infected with P. berghei

Using the Tukey’s multiple comparison test, the percentage parasite clearance was compared between the negative control versus experimental groups. Compared to the negative control, the root bark extracts exhibited significantly higher parasite clearance at 100 mg/kg dose (mean difference (MD) = 15.6, 95%CI = 14.95–16.25; p < 0.001), 200 mg/kg (MD = 16.42, 95%CI = 15.77–17.07; p < 0.001) and 400 mg/kg (MD = 17.24, 95%CI = 16.59–17.89, p < 0.001) (Table 1).

### Efficacy of Z. chalybeum aqueous rootbark extracts on fever level in Wistar albino mice infected with P. berghei

When the mean body temperature of the negative control group and the experimental groups was compared, experimental groups had mean body temperature kept within the normal range. The mean temperature difference was found to be significantly lower among the experimental group as compared to the negative control group which was, MD=−1.50, 95%CI = 2.59–0.67; p = 0.009 at 100mg/kg dose; MD=−1.63, 95%CI = 2.74–0.81; p = 0.004 at 200mg/kg and MD=−1.78, 95%CI = 2.46–0.54; p = 0.002 at 400mg/kg of administered dose (Table 2).

### Efficacy of Z. chalybeum aqueous rootbark extract on the hemoglobin level in Wistar albino mice infected with P. berghei

The findings of the study showed that, the mean hemoglobin concentration across the experimental groups was > 11g/dl in five days. There was no statistical difference between mean haemoglobin level in day one as compared to day five (p > 0.05) as shown in table 3. Further, there was no statistically significant difference in the mean haematocrit concentrations of the experimental group and negative control (p > 0.05) as presented in table 4.

### Effect of Z. chalybeum aqueous rootbark extracts on the survival time of experimental Wistar albino mice infected with P.berghei

**Figure 1:**
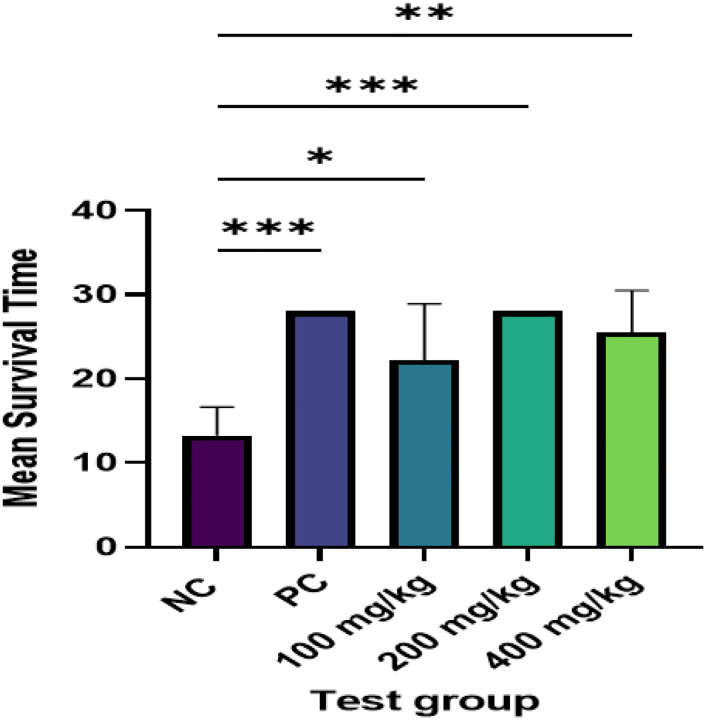
Survival time of *Plasmodium berghei* NK65 infected-mice after treatment with *Zanthoxylum chalybeum* aqueous root bark extracts *Z. chalybeum* rootbark extracts increase mean survival time of *Plasmodium berghei* NK65 infected-mice (p>0.001). *** p<0.001, strong association, ** p<0.01 moderately strong relation, * p<0.05, weak association, NC-Negative Control (10ml/kg), PC-Positive Control (4mg/kg)

The mean survival time between the experimental groups and the negative control was also compared using Tukey’s’ multiple comparison test. The mean survival time in days of mice that received aqueous rootbark extracts of *Z. chalybeum* was 22 days in 100mg/kg, 28days 200mg/kg and 25 days in 400mg/kg while the negative control had mean survival time of 13days. There was a significant association between the use *of Z. chalybeum* rootbark extracts and increase in mean survival time (p > 0.001) as shown in Fig. 1.

## Discussion

In this study, *Z. chalybeum* aqueous rootbark extracts demonstrated parasite clearance up to 76% in Wistar albino mice infected with *P. berghei.* This was achieved at 400mg/kg daily dose. However, the aqueous root bark extracts showed lower parasite clearance than the positive control which had a parasite reduction level of 93% at 10mg/kg daily dose. In this study crude root bark extracts were used. The presence of interacting bioactive compounds in crude extracts may potentially modulate the antimalarial efficacy of *Z. chalybeum* accounting for the reduced parasite clearance as compared to the positive control. However, this study did not investigate the phytochemical composition contributing to the anti-plasmodial activity of the *Z. chalybeum* aqueous crude extracts. Nevertheless, *Z. chalybeum* showed anti-plasmodial potential that presents an opportunity for further studies on its development as conventional antimalarial medicine. The present study findings compare to *in vivo* study results which showed chemo suppressive effects of *Z. chalybeum* aqueous root barks up to 78% in Wistar albino mice [21]. Notably, several studies that reported the anti-plasmodial studies of stem, leaves and rootbarks extracts of *Z. chalybeum* were done *in vitro* [17, 32–34], making it difficult to compare therapeutic outcomes with the present study. Still, these studies also showed that *Z. chalybeum* extracts had good activity against *P. falciparum* at IC50 of 5 μg/ml, 10 μg/ml, and 20 μg/ml respectively [17, 32–34].

Further, crude extracts of *Z. chalybeum* were able to maintain the body temperature of Wistar albino mice within normal of 36°C-37°C as compared with the negative control group. However, there were no remarkable differences between the haemoglobin and haematocrit concentrations of the experimental and negative control groups. The average core temperature of laboratory mice under standard housing conditions is close to that of humans with an average temperature of just about 1°C below resting body temperature [35, 36]. This temperature is highly prone to dysregulation by number of physiological, behavioral and pathological processes. In our experiment, infection with *P. berghei* may have affected the thermoregulatory mechanism in negative experiment group making them unable to maintain their body temperature within the normal range. Our study showed the efficacy of Z. *chalybeum* in maintaining the normal temperature in experimental groups, however the mechanisms underlying the anti-pyretic effects of *Z. chalybeum* were not investigated. Noteworthy, the findings of this study were comparable to an *in vivo* study which showed the anti-pyretic effect of *Z. zanthoxyloids* aqueous stem bark extracts in Winstar albino mice induced inflammation [37].

### Study limitations

This study had some limitations. This study did perform phytochemical screening and quantification of bioactive compounds contributing to the anti-plasmodial activity of the *Z. chalybeum* aqueous crude extracts. Additionally, the anti-pyretic mechanisms of *Z. chalybeum* were not determined.

## Conclusions

*Z. chalybeum* showed efficacy in parasite clearance and maintaining the body temperature of Wistar albino mice infected with *P. berghei*. Additional studies should be done to determine the anti-plasmodial and anti-pyretic mechanisms of *Z. chalybeum* root bark extracts.

## Figures and Tables

**Table 1:** Parasite clearance of *Plasmodium berghei* NK65 infected-mice treated with *Zanthoxylum chalybeum* aqueous rootbark extracts

	Percentage parasite clearance, SD							
Treatment dose	Day 1	Day 2	Day 3	Day 4	Day 5	MD	95%CI	P-value
NC (4ml/kg)	0.00	0.00	0.00	0.00	0.00	1		
PC (10mg/kg)	−0.098 ± 3.11	57.33 ± 2.00	78.65 ± 0.71	89.49 ± 0.77	93.08 ± 0.27	21.07	20.42–21-72	*0.001
**Crude extracts**								
100 mg/kg	−0.19 ± 2.02	50.11 ± 0.54	51.08 ± 0.83	65.87 ± 3.00	68.92 ± 0.79	15.60	14.95–16.25	*0.0001
200 mg/kg	−0.073 ± 2.41	53.84 ± 0.91	62.44 ± 1.69	66.73 ± 1.78	72.54 ± 0.59	16.42	15.77–17.07	*0.0001
400 mg/kg	−0.26 ± 4.48	54.92 ± 0.56	67.71 ± 0.85	72.32 ± 1.51	76.18 ± 0.75	17.24	16.59–17.89	*0.0001

NC-Negative control (distilled water); PC-Positive control (Artemether Lumefantrine); SD-Standard deviation; MD-Mean difference; CI-Confidence interval

* Refers to significant p-value

**Table 2:** Mean body temperature of *Plasmodium berghei NK65* infected-mice treated with *Zanthoxylum chalybeum* rootbark aqueous extracts

	Body temperature (°C), mean (SD)							
Treatment dose	Day 1	Day 2	Day 3	Day 4	Day 5	MD	95%CI	P-value
NC (10 mL/kg)	37.35 ± 0.30	36.65 ± 0.44	35.30 ± 0.72	34.90 ± 0.60	35.75 ± 0.19	1		
PC (4 mg/kg)	37.05 ± 0.38	36.93 ± 0.45	36.93 ± 0.37	37.13 ± 0.52	37.25 ± 0.25	−0.13	2.464–0.536	*0.0018
**Crude extracts**								
100 mg/kg	36.35 ± 0.19	36.08 ± 0.35	36.40 ± 0.33	37.05 ± 0.17	37.25 ± 0.23	−1.50	−2.589–0.661	*0.0009
200 mg/kg	36.13 ± 0.18	36.85 ± 0.41	36.48 ± 0.29	37.48 ± 0.16	37.38 ± 0.17	−1.63	−2.739–0.811	*0.0004
400 mg/kg	36.73 ± 0.11	36.60 ± 0.10	36.65 ± 0.32	37.05 ± 0.42	37.53 ± 0.26	−1.78	−2.464–0.536	*0.0018

NC-Negative control (distilled water); PC-Positive control (Artemether Lumefantrine); SD-Standard deviation; MD-Mean difference; CI-Confidence interval

* Refers to significant p-value

**Table 3:** Haemoglobin level of *Plasmodium berghei NK65* infected mice treated with *Zanthoxylum chalybeum* aqueous rootbark extracts

	Haemoglobin concentration (g/dL), SD							
Treatment dose	Day 1	Day 2	Day 3	Day 4	Day 5	MD	95%CI	P-value
NC (10mL/kg)	12.53 ± 0.51	13.40 ± 0.44	13.45 ± 0.029	13.45 ± 0.38	12.53 ± 0.47	1		
PC (4 mg/kg)	12.98 ± 0.45	13.08 ± 0.27	13.60 ± 0.33	13.13 ± 0.57	12.65 ± 0.22	1.08	−1.366–2.016	0.974
**Crude extracts**								
100 mg/kg	12.28 ± 0.21	13.03 ± 0.63	13.25 ± 0.56	12.00 ± 0.89	11.20 ± 1.05	0.33	−2.109–4.259	0.832
200 mg/kg	13.18 ± 0.43	13.60 ± 0.58	13.65 ± 0.34	13.53 ± 0.085	12.78 ± 0.45	1.75	1.393–2.193	0.956
400 mg/kg	13.25 ± 0.065	13.35 ± 0.62	13.05 ± 0.38	12.78 ± 0.13	12.18 ± 0.52	0.40	0.687–2.837	0.366

NC-Negative control (distilled water); PC-Positive control (Artemether Lumefantrine); SD-Standard deviation; MD-Mean difference; CI-Confidence interval

**Table 4:** Haematocrit concentration of *Plasmodium berghei* NK65 infected-mice treated with *Zanthoxylum chalybeum* aqueous extracts

	Haematocrit concentration (%), SD							
Treatment dose	Day 1	Day 2	Day 3	Day 4	Day 5	MD	95%CI	P-value
NC (10mL/kg)	36.75 ± 1.60	39.50 ± 1.19	39.50 ± 0.29	39.50 ± 1.26	36.25 ± 1.11	1		
PC (4 mg/kg)	38.00 ± 1.29	38.50 ± 0.96	40.00 ± 1.08	38.50 ± 1.66	37.25 ± 0.75	0.75	−4.440–5.940	0.991
**Crude extracts**								
100 mg/kg	36.25 ± 0.63	38.75 ± 1.84	39.00 ± 1.68	35.50 ± 2.60	34.25 ± 3.15	2.00	−7.421–11.420	0.963
200 mg/kg	39.00 ± 1.23	40.00 ± 1.68	40.00 ± 1.08	39.75 ± 0.25	37.25 ± 1.44	1.75	−3.643–7.143	0.850
400 mg/kg	39.00 ± 0.00	39.25 ± 1.70	38.25 ± 1.11	37.50 ± 0.50	35.75 ± 1.65	3.25	−1.955–8.455	0.345

NC-Negative control (distilled water); PC-Positive control (Artemether Lumefantrine); SD-Standard deviation; MD-Mean difference; CI-Confidence interval

## Data Availability

The data sets used and/or analyzed during this study are available from the corresponding author upon request.

## Abbreviations

ACT: Artemisinin-based Combination Therapy
MUST: Mbarara University of Science and Technology
UNCST: Uganda National Council of Science and Technology
LD50: Lethal Dose at 50
OECD: Organisation of Economic Cooperation and Development
Hb: Haemoglobin
HCT: Haematocrit
MST: Mean Survival Time
ANOVA: Analysis of Variance
MD: Mean Difference
CI: Confidence Interval
WHO: World Health Organisation
